# Inhibition of breast cancer cell motility with a non-cyclooxygenase inhibitory derivative of sulindac by suppressing TGFβ/miR-21 signaling

**DOI:** 10.18632/oncotarget.6888

**Published:** 2016-01-12

**Authors:** Bin Yi, Hong Chang, Ruixia Ma, Xiangling Feng, Wei Li, Gary A. Piazza, Yaguang Xi

**Affiliations:** ^1^ Mitchell Cancer Institute, University of South Alabama, Mobile, AL, USA; ^2^ School of Public Health, Central South University, Changsha, Hunan, China; ^3^ Department of Pharmaceutical Science, University of Tennessee Health Science Center, Memphis, TN, USA

**Keywords:** sulindac, breast cancer, metastasis, microRNA, TGFβ

## Abstract

Compelling efficacy on intervention of tumorigenesis by nonsteroidal anti-inflammatory drugs (NSAIDs) has been documented intensively. However, the toxicities related to cyclooxygenase (COX) inhibition resulting in suppression of physiologically important prostaglandins limit their clinical use for human cancer chemoprevention. A novel derivative of the NSAID sulindac sulfide (SS), referred as sulindac sulfide amide (SSA), was recently developed, which lacks COX inhibitory activity, yet shows greater suppressive effect than SS on growth of various cancer cells. In this study, we focus on the inhibitory activity of SSA on breast tumor cell motility, which has not been studied previously. Our results show that SSA treatment at non-cytotoxic concentrations can specifically reduce breast tumor cell motility without influencing tumor cell growth, and the mechanism of action involves the suppression of TGFβ signaling by directly blocking Smad2/3 phosphorylation. Moreover, miR-21, a well-documented oncogenic miRNA for promoting tumor cell metastasis, was also found to be involved in inhibitory activity of SSA in breast tumor cell motility through the modulation of TGFβ pathway. In conclusion, we demonstrate that a non-COX inhibitory derivative of sulindac can inhibit breast tumor metastasis by a mechanism involving the TGFβ/miR-21 signaling axis.

## INTRODUCTION

Non-steroidal anti-inflammatory drugs (NSAIDs) are commonly used to treat a variety of inflammatory conditions and pain associated with arthritis. Previous studies have reported promising cancer chemopreventive activity from regular use of aspirin and other NSAIDs for a variety of malignancies including breast cancer, colon cancer, and other solid tumors [1–4]. In particular, the NSAID, sulindac has been shown to reduce the size and number of precancerous adenomas in patients with familial or sporadic adenomatous polyposis [5–7]. Cyclooxygenase-2 (COX-2) inhibition is often regarded as the pharmacological basis responsible for the anti-inflammatory properties of NSAIDs, which is also considered to be the mechanism responsible for the cancer chemopreventive activity of NSAIDs [8, 9]. Unfortunately, the long-term use of NSAIDs for cancer chemoprevention is not recommended because of gastrointestinal, renal, or cardiovascular toxicities associated with the depletion of physiologically important prostaglandins resulting from COX-2 inhibition [10, 11]. However, different studies have also reported that distinct mechanisms from COX inhibition could be fully or partially responsible for the cancer chemoprevention activity of NSAIDs [12–15].

Breast cancer remains the second leading cause of death among women in the United States [16]. According to American Cancer Society, the 5-year survival rate for patients with non-metastatic breast cancer is 99%; whereas the rate is dramatically reduced to 25% for the individuals with distant metastases [17]. Therefore, effective and safe chemopreventive agents to inhibit tumor progression and metastasis are urgently needed in the clinic. With regard to breast cancer, epidemiological studies have reported that NSAIDs can significantly reduce breast cancer incidence, recurrence and the risk of death [1, 2]. For example, a large population-based study that was conducted by the Women's Health Initiative followed 80,741 post-menopausal women for more than 8 years and determined that the long-term use of aspirin can reduce breast cancer risk by 21% [1]. A recent study also reported that the daily use of aspirin could reduce the risk of death from solid tumor metastasis by up to 48% based on meta-analyses of a large number of randomized controlled clinical trials [3]. These studies highlight important anti-cancer activities of NSAIDs not only to prevent tumor progression, but also to suppress metastasis. The tumor cell growth inhibitory activity of NSAIDs is considered to partially attribute to their ability in prevention of tumor progression, while the mechanism responsible for their suppressive effect on tumor metastasis is largely unknown.

Sulindac sulfide amide (SSA) is a recently characterized amide derivative of the NSAID, sulindac sulfide (SS). It has no COX inhibitory properties, but shows improved potency to inhibit colon tumor cell growth both *in vitro* and *in vivo* [18]. Here, by studying the anti-metastatic activity of SSA, for the first time, we report that SSA can inhibit motility of a panel of breast tumor cells at concentrations less than those required to inhibit tumor cell growth. The mechanism of action involves suppression of TGFβ signaling by directly blocking the phosphorylation of Smad2/3. Moreover, miR-21, a well-documented oncogenic miRNA for promoting tumor cell metastasis, was also found to be involved in the inhibitory activity of SSA in breast tumor cell motility through the modulation of TGFβ pathway. Therefore, our results provide novel evidence of anti-metastatic activity for a non-COX inhibitory derivative of sulindac, SSA in breast cancer and demonstrate that the mechanism of action involves suppression of the TGFβ/miR-21 pathway.

## RESULTS

### SSA inhibits tumor cell motility at sub-cytotoxic concentrations

**Figure 1:**
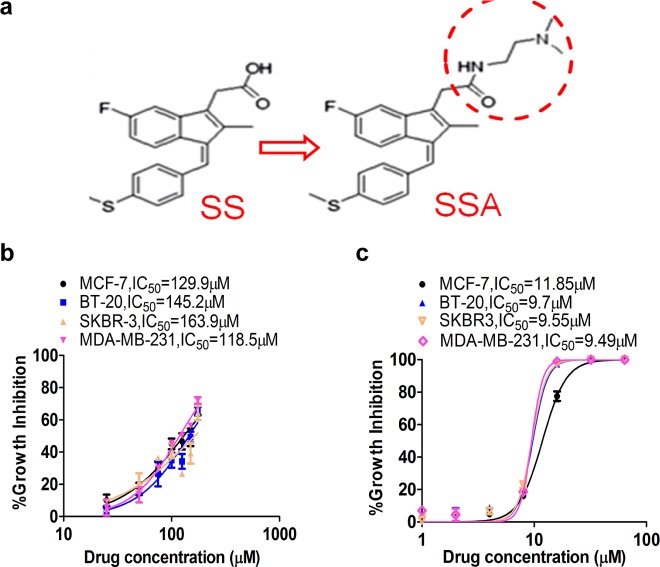
SSA shows greater potency to inhibit breast cancer cell growth compared to SS **a.** The chemical structure schemes of SS and SSA. **b.** Breast cancer MCF-7, MDA-MB-231, BT-20, and SKBR-3 cells were treated with SS at 25, 50, 75, 100, 125, 150, and 175 μM for 36 h. **c.** Breast cancer MCF-7, MDA-MB-231, BT-20, and SKBR-3 cells were treated with SSA at 1, 2, 4, 8, 16, 32, and 64 μM for 36 h. Cell growth inhibitory activity was evaluated by using Cell Titer-Glo Assay, which measures viable cell numbers based on ATP content. The relative cell viability was computed and the growth inhibition curve was plotted in which IC_50_ was calculated by using GraphPad Prism 6.

**Figure 2:**
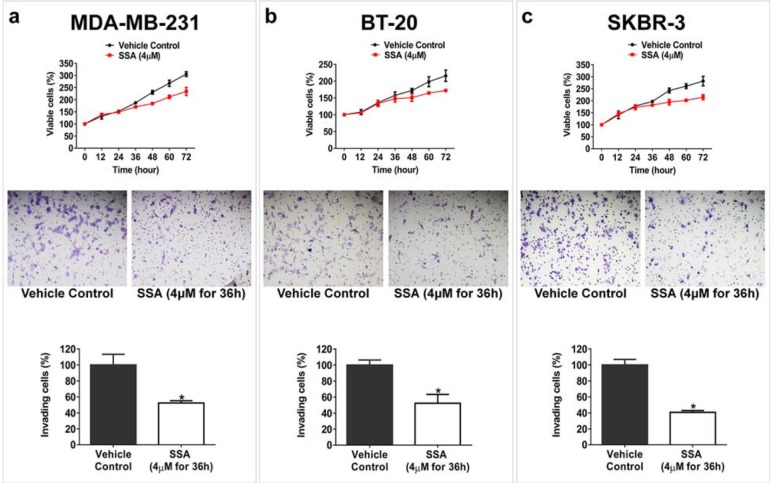
SSA inhibits breast tumor cell invasion at a sub-cytotoxic condition Upper panels: **a.** MDA-MB-231, **b.** BT-20, and **c.** SKBR-3 cells were treated with 4 μM SSA at different time points; the viability of these cells were not significantly affected prior to 36 h (*P* > 0.05). Middle panels: The inhibitory effect of SSA (4 μM for 36 h) on invasion of (a) MDA-MB-231, (b) BT-20, and (c) SKBR-3 cells were evaluated by using BD Matrigel invasion assay. After removing the non-invading cells with a clean cotton swab, the invading cells were fixed with formaldehyde and then stained with crystal violet before counting. Bottom panels: Quantification analyses of SSA inhibitory effect on breast tumor cell invasion. Five microscopic fields randomly chosen from each assay were counted individually, and the statistical significance between SSA treatment and the vehicle control was determined by t-test (*P < 0.05).

SSA is an amide derivative of SS lacking COX inhibitory properties but with potent tumor cell growth inhibitory activity compared with SS [18]. The chemical structure of SSA and SS are shown in Figure 1a to illustrate the substitution of the carboxylic acid with a dimethylethyl amide moiety. A panel of breast cancer cells, including MCF-7, BT-20, SKBR-3, and MDA-MB-231 cells was employed in this study to investigate the anti-metastastic activity of SSA. First, the cytotoxicity of SS and SSA was determined after 36 h of treatment. The results showed that the growth inhibitory potency of SSA was approximately 10 times greater than SS in all four breast tumor cell (Figure 1b and 1c). Using a protocol as reported previously [19], we determined the effect of non-cytotoxic concentrations of SSA on tumor cell invasion, and we found that SSA treatment at 4 μM for 36 h significantly inhibited the invasion of highly metastatic breast cancer cell lines, MDA-MB-231, BT-20, and SKBR-3 (Figure 2). We also studied the inhibitory effect of SSA (4 μM, 36 h) on tumor cell migration in the same cell lines by using a wound-healing assay, which showed similar inhibitory activity (Supplementary Figure S1). These data demonstrate that SSA can inhibit breast tumor cell invasion and migration at non-cytotoxic concentrations; whereas we previously reported that SS has similar activity on breast and colon tumor cells but at a concentration (50 μM) over 10 times higher than SSA [19].

### Anti-invasive activity of SSA involves suppression of TGFβ signaling

**Figure 3:**
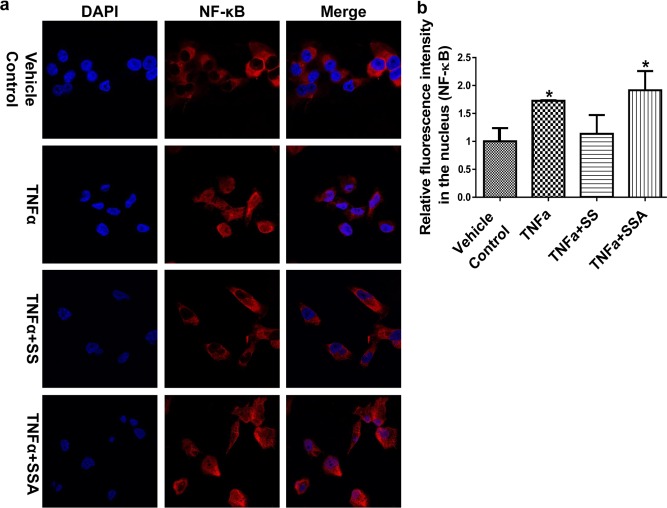
SSA does not influence the nuclear translocation of NF-κB in MDA-MB-231 cells **a.** The immunofluorescence imaging results show that SS can block NF-κB to enter the nucleus by attenuating TNFα simulative effect in MDA-MB-231 cells, but SSA doesn't. The treatments include the vehicle control (0.1% DMSO), TNFα (25 ng/ml), TNFα+SS (50 μM), and TNFα+ SSA (4 μM). Red: NF-κB antibody; Blue: DAPI. Images were captured by using Nikon Eclipse Ti Laser Confocal Scanning Microscopy.**b.** Quantitative analysis of NF-κB in the nucleus. The relative fluorescent intensity of NF-κB was analyzed by using NIS-Elements AR imaging software. Data are presented as the mean of thirty measurements ± standard deviation. *P < 0.05.

We previously reported that the blockade of NF-κB signaling by SS is one of the key mechanisms associated with its anti-invasive activity [19]. Hence, we are interested in examining if the same mechanism of action is involved in SSA inhibitory activity as well. By employing the NF-κB immunofluorescence assay, we treated MDA-MB-231 cells with TNFα to induce the translocation of NF-κB to the nucleus. Then SS and SSA were added at non-cytotoxic concentrations. As shown in Figure 3, SS, but not SSA, attenuated the inductive effect of TNFα on accumulation of nuclear NF-κB. These results suggest a distinct mechanism from suppression of NF-κB is responsible for SSA anti-invasive activity, which may be consistent with its inability to inhibit COX activity.

**Figure 4:**
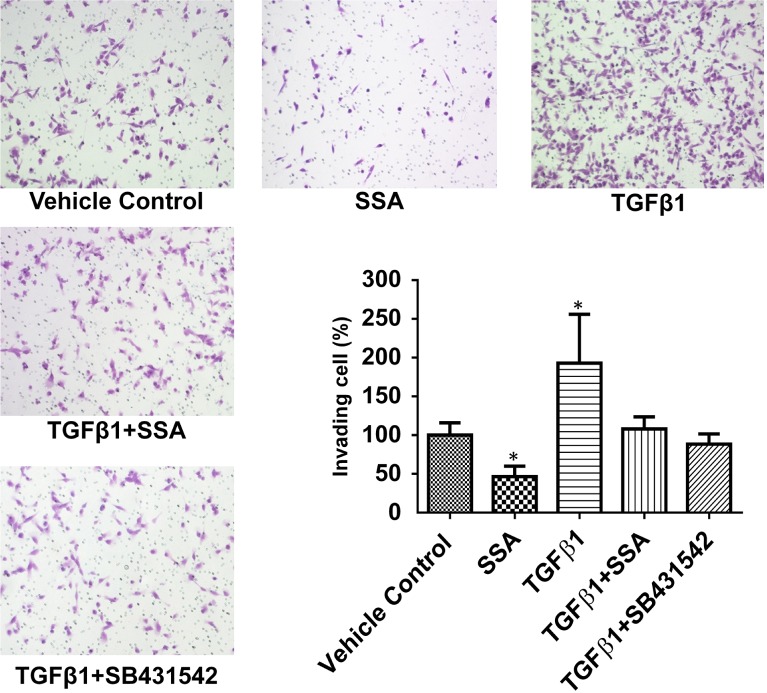
SSA attenuates the inductive effect of TGFβ1 on invasion of MDA-MB-231 cells BD Matrigel invasion assay was used to evaluate the motility of MDA-MB-231 cells with different treatments including the vehicle control (0.1% DMSO), SSA (4 μM), TGFβ1 (30 ng/ml), TGFβ1+SSA, and TGFβ1+SB431542 (TGFβ1 receptor inhibitors, 10 μM). Five microscopic fields randomly chosen from each assay were counted individually, and the statistical significance between different treatments and vehicle control was determined by t-test. *P < 0.05.

Multiple studies have reported that TGFβ signaling plays an important role in tumor progression and metastasis [20–22]. To determine if inhibition of TGFβ signaling mediates that anti-invasive activity of SSA, MDA-MB-231 cells were treated with TGFβ1 at a concentration of 30 ng/ml to induce cell motility prior to the post-treatment with SSA at 4 μM. As measured by BD matrigel cell invasion assay, SSA was found to attenuate the stimulatory effect of TGFβ1 on cell motility (Figure 4). Similarly, the TGFβ1 receptor inhibitor, SB431542, inhibited TGFβ1 stimulation of tumor cell motility to an extent comparable to SSA. These results suggest that SSA inhibits breast tumor cell invasion by suppressing TGFβ receptor-mediated signaling.

**Figure 5:**
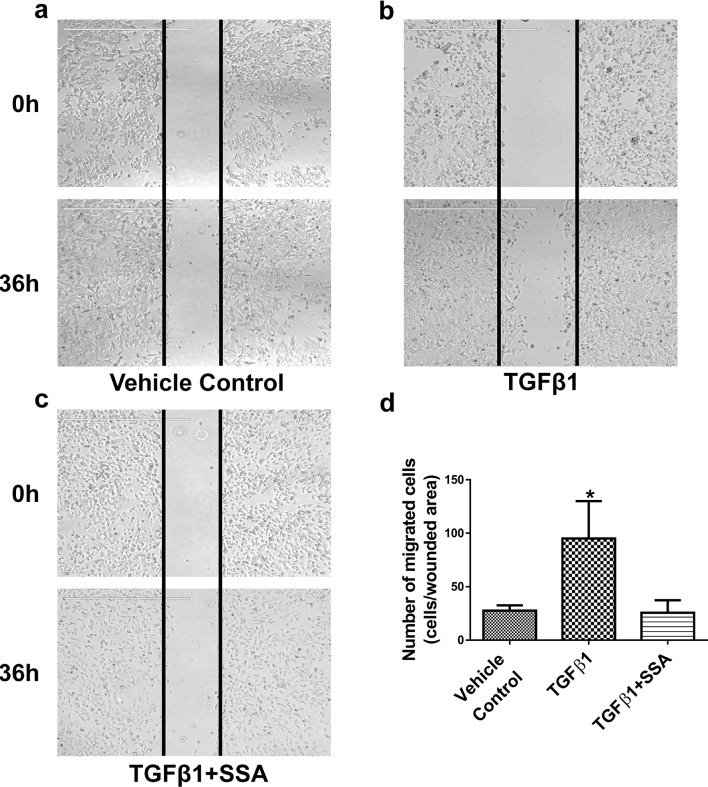
SSA inhibits MCF-7 cell motility induced by TGFβ1 as evaluated by a wound-healing assay After scraping a straight line on the surface of pre-seeded cells with using a sharp pipette tip, MCF-7 cells were treated with **a.** the vehicle control (0.1% DMSO), **b.** TGFβ1 (30 ng/ml), **c.** TGFβ1 + SSA (4 μM), for 36 h, respectively. The cell motility was evaluated by using EVOS FL Cell imaging System, and migrating cells in the wound areas were counted. **d.** Quantification of tumor cell motility was exhibited with the average numbers of migrating cells ± standard deviation in three independent experiments. *P < 0.05.

To further study this possibility, MCF-7 breast tumor cells, a well-known non-invasive breast tumor cell model, were treated with 30 ng/ml TGFβ1 for 36 h to induce tumor cell motility by referring to the published protocol [23, 24]. As shown in Figure 5, TGFβ1 significantly stimulated motility of MCF-7 cells as measured by the wound-healing assay; whereas SSA treatment at 4 μM effectively attenuated the stimulatory effect of TGFβ1. These results further support that SSA inhibition of breast tumor cell motility involves the suppression of TGFβ signaling.

### SSA suppresses TGFβ signaling by blocking phosphorylation of Smad2/3

**Figure 6:**
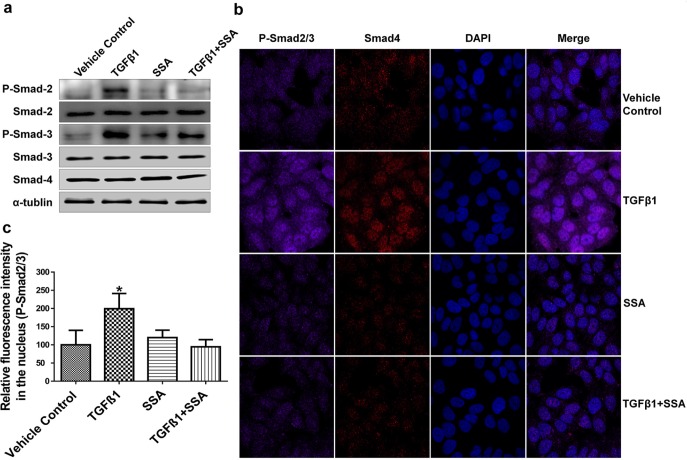
Inhibition of Smad-2/3 phosphorylation is responsible for SSA Suppression of TGFβ signaling **a.** SSA inhibits phosphorylation of Smad-2/3 in MCF-7 cells by Western blotting; **b.** SSA reduces the signals of phosphorylated Smad2/3 in MCF-7 cells by immunofluorescence imaging. SSA at 4 μM and TGFβ1 at 30ng/ml were used to treat cells. Violet: p-Smad2/3, Red: Smad4, and Blue: DAPI. **c.** Quantitative analysis of phosphorylated Smad2/3 fluorescent signals in the nucleus. The relative fluorescent intensity was analyzed by using NIS-Elements AR imaging software. Data are graphed as the mean of thirty measurements ± standard deviation. *P < 0.05.

To further define the mechanism by which SSA inhibits TGFβ signaling, the phosphorylation of Smad2 and Smad3 was measured in MCF-7 breast tumor cells following the treatment with TGFβ1. The translocation and accumulation of the complex of phospho (p)-Smad2/3 and Smad4 in nucleus are key events for transduction of TGFβ receptor-mediated signaling [25]. As shown in Figure 6a, SSA significantly reduced the phosphorylation of Smad2 and Smad3, and attenuated the inductive effect of TGFβ1 treatment without interruption of the expression of total Smad2, Smad3, and Smad4. In addition, the expression levels of p-Smad2, p-Smad3, and Smad4 in the nucleus of MCF-7 breast tumor cells were measured by immunofluorescence microscopy, and we found that SSA could reduce the level of p-Smad2/3 in the nucleus with or without the presence of TGFβ1(Figure 6b). These results suggest that the mechanism by which SSA inhibits TGFβ signaling involves inhibition of Smad2 and Smad3 phosphorylation.

### MiR-21 is involved in anti-invasive activity of SSA when targeted by TGFβ/Smad2/3

**Figure 7:**
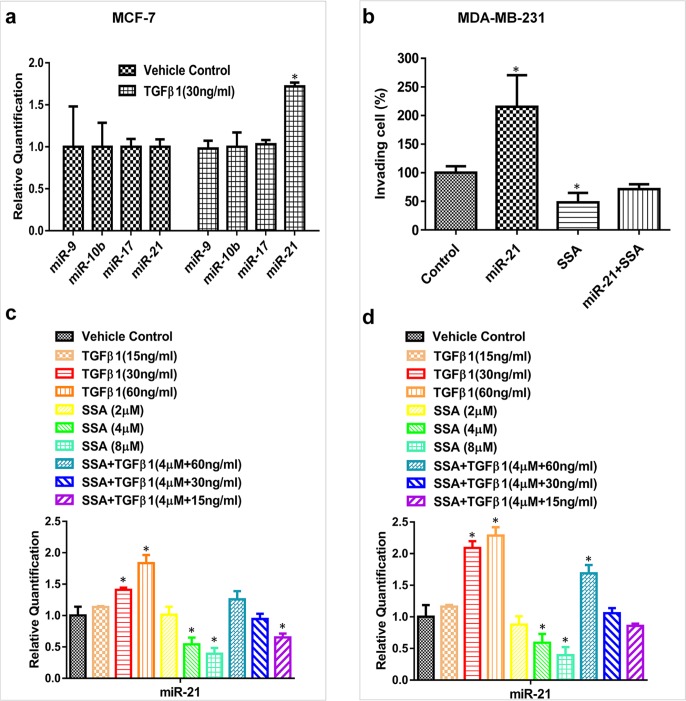
SSA regulates miR-21 expression through the blockade of TGFβ signaling **a.** TGFβ1 at 30 ng/ml can induce miR-21 but not miR-10b, 17, and -9 expressions in MCF-7 cells as measured by qRT-PCR. **b.** SSA at 4 μM can inhibit the cell invasion induced by miR-21 mimics in MDA-MB-231 cells. BD Matrigel invasion assay was used to quantitate the motility of MDA-MB-231 cells in which the vehicle control or miR-21 mimics were transiently transfected. The transfected cells were also treated with SSA at 4 μM to observe its inhibitory effect on the invasive phenotype induced by miR-21. **c.** The combinations of SSA and TGFβ1 at different concentrations can alter the expression of miR-21 variably in MCF-7 cells as evaluated by qRT-PCR. **d.** The combinations of SSA and TGFβ1 at different concentrations can alter the expression of miR-21 variably in MDA-MB-231 cells as evaluated by qRT-PCR. *P < 0.05.

MiR-10b, miR-17, miR-21, and miR-9 are oncogenic miRNAs that have been well-documented to promote tumor cell invasion and metastasis in numerous studies [26–29]. We previously reported that SS downregulates these miRNAs by suppressing NF-κB signaling [19]. In this study, we are interested in determining if these miRNAs are also involved in anti-invasive activity of SSA. In MCF-7 cells showing increased motility after TGFβ1 treatment, we examined the expression of miR-10b, miR-17, miR-21, and miR-9, but found only miR-21 was upregulated (Figure 7a). We therefore studied the influence of SSA on miR-21 expression in metastatic MDA-MB-231 cells. As shown in Figure 7b, overexpression of miR-21 by transfection of its mimics can increase the invasion of MDA-MB-231 cells; while SSA not only reduced cell invasion, but also attenuated the promoting effect of miR-21. These results suggest that miR-21 can mediate the inhibitory effect of SSA on breast tumor cell invasion and that the effects of SSA appear to be more specific when compared with SS.

To further study the involvement of miR-21, MCF-7 and MDA-MB-231 cells were treated with different concentrations of TGFβ1 in the presence or absence of SSA, and the expression of miR-21 was measured by qRT-PCR. As shown in Figure 7c and 7d, TGFβ1 upregulated miR-21 expression in a dose-dependent manner, while SSA attenuated the inductive effect of TGFβ1 on the expression of miR-21. The interaction between SSA and TGFβ to regulate miR-21 expression was further validated by using SSA and TGFβ1 at various concentrations as well as their combinations. Obviously, higher concentrations of TGFβ1 show more significant attenuation on SSA suppression of miR-21. These results suggest that downregulation of miR-21 by SSA might be through modulation of TGFβ signaling.

**Figure 8:**
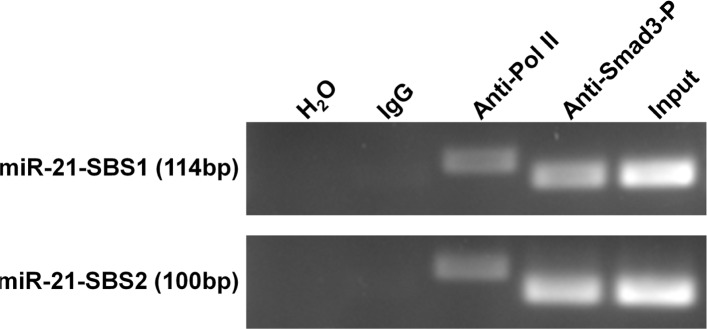
ChIP assay demonstrates the direct binding of p-Smad3 on the promoter of miR-21 gene MCF-7 cells were pre-treated with 30 ng/ml TGFβ1 for 1 hour and immunoprecipitated by p-Smad3 antibody, RNA polymerase II antibody, or normal mouse IgG. The isolated DNA fragments were used for the templates in PCR analysis. SBS1 (ATGCATTCT) and SBS2 (AAGTCAGAGAG) are the reported binding sequences of p-Smad3. RNA polymerase II binding GAPDH promoter was used as a positive control system to confirm the efficiency of ChIP assay. The lengths of the PCR products including SBS1, SBS2, and GAPDH promoter sequences are 114bp, 100bp, and 166 bp, respectively.

We next used a ChIP assay to determine the regulation of miR-21 expression by TGFβ. In the promoter of miR-21, we identified two binding sites of p-Smad3, SBS1 (ATGCATTCT) and SBS2 (AAGTCAGAGAG), as reported previously [30, 31]. Then, the sheared chromatin from MCF-7 cells pretreated with TGFβ1 was precipitated by using anti-phosphorylated Smad3 antibody. After isolated DNA fragments from the pull-down complex, we performed PCR to amplify the target fragments including SBS1 and SBS2 sequences. As shown in Figure 8, the expected bands were found, demonstrating that the DNA fragments immunoprecipitated by the anti-phosphorylated Smad3 antibody contain the promoter sequences of miR-21.

**Figure 9:**
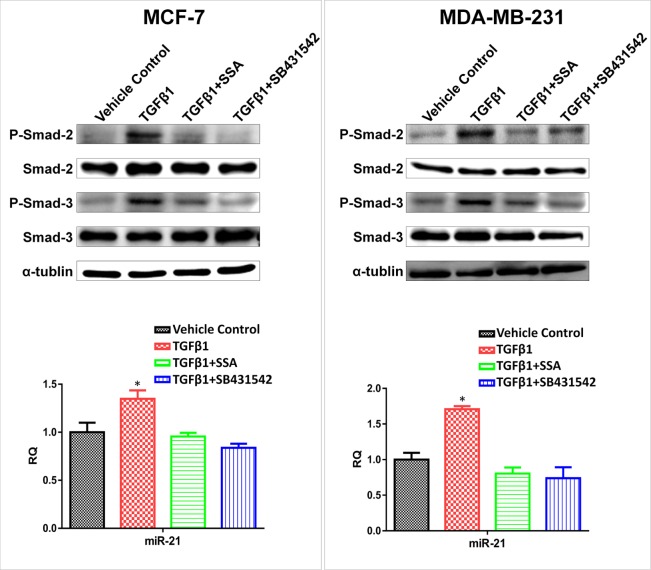
Downregulation of miR-21 by SSA is through repression of the phosphorylation of Smad2/3 Upper panels: SSA (4 μM) and SB431542 (10 μM) can attenuate the inductive effect of TGFβ1 (30 ng/ml) on phosphorylation of Smad-2/3 in MCF-7 and MDA-MB-231 cells as determined by Western blotting. Bottom panels: Blockade of TGFβ signaling by SSA or SB431542 can lead to downregulation of miR-21 in MCF-7 and MDA-MB-231 cells as determined by qRT-PCR. *P < 0.05.

To further study the interaction between SSA and TGFβ signaling in regulation of miR-21 expression, MCF-7 and MDA-MB-231 breast tumor cells were treated with 4 μM SSA, 30 ng/ml TGFβ1, and their combination, respectively. The TGFβ1 receptor inhibitor, SB431542 was included as the control. As shown in Figure 9, TGFβ1 treatment induced the phosphorylation of Smad2/3, which in turn, led to upregulation of miR-21 in both breast tumor cell lines; whereas SSA reduced miR-21 expression through inhibition of Smad2/3 phosphorylation. SB431542 showed a similar phenotype to SSA, which demonstrates the direct inhibition of Smad2/3 phosphorylation by SSA. These results show that the transcriptional regulation of TGFβ on miR-21 expression depends on the direct binding of p-Smad2/3 to the promoter regions of miR-21.

## DISCUSSION

Previous studies have documented that long term administration with NSAIDs can reduce the risk of death and malignant recurrence in breast cancer patients [1, 2]. However, their use for cancer chemoprevention is not recommended because of potentially fatal toxicities resulting from inhibition of cyclooxygenase (COX) and suppression of prostaglandins synthesis [10, 11]. SSA is a novel amide derivative of SS, which lacks COX inhibitory activity, yet displays potent growth inhibitory activity against colon tumor cells [18]. SSA was previously reported to have anti-tumor activity in the HT29 colon tumor xenograft mouse model and the TRAMP mouse model of prostate tumorigenesis [18, 32]. The mechanism responsible for its tumor cell growth inhibitory activity involves the induction of autophagic cell death by suppressing Akt/mTOR signaling as recently reported in lung adenocarcinoma cell lines [33]. However, there are no studies that have investigated the anti-metastatic activity of SSA in human cancers. Given its potential for greater anticancer efficacy and reduced toxicity as compared to sulindac, we studied the possibility that SSA inhibits tumor cell metastasis and investigated the underlying mechanism. By evaluating a panel of highly aggressive human breast tumor cell lines, including MDA-MB-231, BT-20, and SKBR-3 cells, SSA was found to significantly inhibit tumor cell growth with more than 10 times greater potency compared to SS. Impressively, SSA was found to inhibit tumor cell invasion and migration at sub-cytotoxic concentrations and was at least 10 times more potent than SS compared to our previous observation [19]. Given that SSA is chemically identical to SS with the exception of the carboxylic acid moiety that is necessary for COX binding, these results provide insight into novel COX-independent mechanisms responsible for the anti-metastatic activity of sulindac and support the feasibility of developing safer and more efficacious derivatives for breast cancer patients with malignant disease.

We previously reported that SS can inhibit the invasion of human breast and colorectal tumor cells through suppressing NF-κB-mediated transcription of a panel of oncogenic miRNAs [19]. However, in this study, we found that SSA was appreciably less effective than SS to inhibit NF-κB signaling. Given recent studies reporting that TGFβ signaling plays an important role in tumor progression and metastasis [20–22], we examined the ability of SSA to inhibit TGFβ signaling using the human metastatic breast tumor cell line, MDA-MB-231. Our results showed that SSA not only inhibited the invasion of MDA-MB-231 cells, but also attenuated the stimulatory effect of TGFβ on tumor cell motility. Using previously published protocols [23, 24], we induced migration of a non-metastatic human breast tumor cell line, MCF-7 by TGFβ1. Intriguingly, SSA showed compelling inhibitory effects on migration induced by TGFβ1. These results lead us to conclude that the blockade of TGFβ signaling is involved in the inhibitory effect of SSA on breast tumor cell motility.

TGFβ binds to specific receptors on the cell membrane, leading to the formation of the complex consisting of phosphorylated Smad2/3 and Smad4. This complex can enter the nucleus where it regulates target gene expression at the transcriptional level [34]. Our results show that SSA can inhibit the phosphorylation of Smad2/3 without interrupting total Smad2/3/4 levels in breast cancer cells. In the recent studies, the NSAID, 5-aminosalicylic acid (5-ASA) was reported to inhibit TGFβ signaling by blocking phosphorylated Smad2/3 to enter the nucleus in colorectal cancer cells [35]; and tolfenamic acid was found to inhibit the phosphorylation of Smad2/3 by interacting with the MAP kinase pathway [36]. These results suggest that the disruption of Smad2/3 phosphorylation may be a key mechanism responsible for the anti-cancer activity of NSAIDs.

MiRNAs have been well documented as “master” regulators of gene expression, and are broadly involved in normal and pathological processes including tumorigenesis 37, 38]. For example, miR-10b, miR-17, miR-21, and miR-9, are considered as oncogenic miRNAs because of their association with tumor cell proliferation and metastasis [26–29]. We recently reported that SS can inhibit breast and colorectal tumor cell invasion by downregulating these oncogenic miRNAs through the modulation of NF-κB signaling [19]. In this study, we found that SSA only alters the expression of miR-21 through a distinct pathway, TGFβ. MiR-21 has been documented for its oncogenic role in malignant transformation, invasion and metastasis [39], and its elevation was found to correlate with lymph node metastasis in patients with breast cancer [40]. The mechanism by which miR-21 promotes tumor invasion and metastasis is associated with its ability to repress multiple tumor suppressor genes, including tropomyosin 1 (TPM1), programmed cell death 4 (PDCD4), and maspin [41]. Previous reports showed that TGFβ1 can induce miR-21 by promoting the processing of the Smad2/Drosha complex, or enhancing the biogenesis of miR-21 through Smad3/4 mediated transcription [30, 42, 43]. In this study, we show that phosphorylated Smad2/3 can directly bind to the promoter of the miR-21 gene to upregulate its expression through the transcriptional control. In addition, we found that TGFβ1 can induce miR-21 expression in a dose-dependent manner, while SSA significantly attenuated TGFβ1-induced miR-21 expression in breast tumor cells.

Small molecule inhibitors of TGFβ1 receptor have been reported to reduce the oncogenic activity of TGFβ1, such as epithelial to mesenchymal transition and tumor metastasis [44, 45]. For example, SB-431542 was shown to suppress tumor progression by inhibiting the transcriptional activity of p-Smad2/3 [46]. In this study, we found that SSA could mimic the inhibitory effect of SB-431542 on phosphorylation of Smad2/3 in both MCF-7 and MDA-MB-231 breast tumor cells. In addition, SSA countered TGFβ1 upregulation of miR-21 expression by inhibiting phosphorylation of Smad2/3, which is similar to SB-431542. Therefore, our results support that downregulation of miR-21 is responsible for the inhibitory effect of SSA on breast tumor cell motility through blockade of TGFβ signaling.

This is the first study to demonstrate the anti-metastatic activity of SSA in human cancer. SSA is the non-COX inhibitory derivative with improved efficacy and potency when compared to its parent compound, SS. Our study demonstrates that SSA can inhibit the phosphorylation of Smad2/3 and thereby impede the transduction of the oncogenic TGFβ signaling into the nucleus. MiR-21 is known to promote tumor metastasis, and we find that miR-21 can be downregulated by SSA through modulating TGFβ signaling. In conclusion, our results not only support the pronounced inhibitory effect of SSA on breast tumor metastasis, but also demonstrate that TGFβ/miR-21 pathway is involved in its mechanism of action. These observations provide critical insight into development of new sulindac derivatives with improved efficacy and reduced toxicity to inhibit tumor progression and metastasis.

## MATERIALS AND METHODS

### Cell culture and compounds

The human breast cancer cell lines, MCF-7, BT-20, SKBR-3, and MDA-MB-231, were purchased from ATCC (Manassas, VA, USA). The Dulbecco's Modified Eagle Medium (MCF-7 and MDA-MB-231 cells), Minimum Essential Medium (BT-20 cells), and McCoy's 5a Medium (SKBR-3 cells) were purchased from Life Technologies (Carlsbad, CA, USA), and used for tissue culture after mixing with 10% fetal bovine serum (Atlanta Biologicals, Lawrenceville, GA, USA) The cells were maintained in 37°C and 5% CO_2_ humidified atmosphere. SSA was synthesized in Dr. Piazza's lab as previously reported [18]; SS was purchased from Sigma-Aldrich (St Louis, MO, USA); TGFβ1 was purchased from PromoCell GmbH (Heidelberg, Germany); and TGFβ1 receptor inhibitor, SB431542, was purchased from Selleckchem (Houston, TX, USA).

### Growth inhibition assay

Cell growth inhibitory activity was examined using Cell Titer-Glo Assay (Promega, Madison, WI, USA), which measures viable cells based on ATP contents. In brief, MCF-7, BT-20, SKBR-3, and MDA-MB-231 cells were seeded in 96-well plates at a density of 5,000 cells per well and incubated for 12 h before the treatments. Then, cells were treated with 25, 50, 75, 100, 125, 150, and 175 μM SS or 1, 2, 4, 8, 16, 32, and 64 μM SSA and incubated for an additional 36 h or treated with 4 μM SSA for 12, 24, 36, 48, 60, and 72 h. At the end of the incubation, the relative cell viability was computed and the growth inhibition curve was plotted in which IC_50_ was calculated by using GraphPad Prism 6 (GraphPad Software, CA, USA).

### Invasion assay

Cell invasion was measured by the Biocoat matrigel invasion chamber kit from BD Bioscience (Sparks, MD, USA) by following the manufacturer's instruction. In brief, cells were suspended in 500 μl blank medium and incubated for 12 h. Then, the matrigel coated plates were rehydrated with warm serum free medium for 2 h. After removing the medium, 2.5 × 10^4^ cells in 500 μl blank medium were added to the upper chamber, and then 750 μl chemoattractant containing 10% FBS was added to the lower compartment. Cells were then incubated in 5% CO_2_ atmosphere at 37°C for 36 h. After removing non-invading cells by using a clean cotton swab, invading cells were fixed with formaldehyde and then stained with crystal violet before counting. For each assay, five randomly chosen microscopic fields were counted and the average of these numbers was recorded.

### Wound-healing assay

Each 2 × 10^5^ cells were seeded in a single well of 6-well plates and cultured until near confluence (85-95%). After serum starvation for 24 h, a sharp pipette tip was used to scrape a straight line in the middle of the well and the floated cells were washed away with warm PBS. Then the cells are maintained at 37°C for observation of migration. For SSA mediated effects, cells were treated with 4 μM SSA, 30 ng/ml TGFβ1, vehicle control (0.1% DMSO) or incubated simultaneously with 30 ng/ml TGFβ1 and 4 μM SSA for 36 h. The cell migration imaging was photographed at 0, 12, 24, 36 h using EVOS^®^ FL Cell imaging System (Fisher Scientific, Pittsburg, PA) after scraping. Migrating cells in the wound area were counted for quantification. Data are presented as the average number of migrating cells ± standard deviation in three independent experiments.

### Western-blot assay

Cells were lysed by RIPA buffer (Sigma-Aldrich) and quantitated with the DC protein assay kit (Bio-Rad, Hercules, CA, USA). For detection of phosphorylation, the samples were kept on ice at all times after adding the cocktail of protease inhibitors (Fisher Scientific) and phosphatase inhibitors (Roche, Indianapolis, IN, USA). The cell lysis containing denatured total proteins were separated on a 10% SDS-PAGE gel and then transferred to PVDF membranes (Bio-Rad). The membrane was blocked with 5% BSA-TBST (Bio-Rad) for detection of phosphorylation or 5% non-fat milk-TBST and then incubated with mouse anti-human α-tubulin monoclonal antibody (Santa Cruz, CA, USA), rabbit anti-human Smad-2 polyclonal antibody (Cell Signaling, Beverly, MA, USA), rabbit anti-human Smad-3 polyclonal antibody (Cell signaling), rabbit anti-human phosphorylated Smad-2 polyclonal antibody (Cell signaling), rabbit anti-human phosphorylated Smad-3 polyclonal antibody (Cell signaling), and rabbit anti-phospho-Smad-2-(Ser465/467)/Smad-3-(Ser423/425) antibody (Cell signaling) at 4°C overnight. After washing with TBST (Bio-Rad), peroxidase linked secondary goat anti-mouse IgG or goat anti-rabbit IgG antibodies (Bio-Rad) were incubated with blots for 1 h at room temperature. After incubating with enhanced chemiluminescent substrate (Thermo Scientific, Worcester, MA, USA), the imaging were visualized by G:BOX Chemi Imager (Syngene, Cambridge, England).

### Real-time PCR

Total RNA was extracted by TRIzol® (Life Technologies) and cDNA was synthesized by a high capacity cDNA reverse transcriptase kit (Life Technologies). The stem-loop RT primers of miRNAs were designed following the previous publication [47]. The 20 μl mixture of reverse transcription reaction included 2 μg of total RNA, 2 μM reverse transcription primers, 2 μl 10× reverse transcription buffer, 0.8 μl 100 mM dNTP and nuclease-free water. The reverse transcription reaction was performed at 37°C for 2 h. The 20 μl mixture of quantitative real-time PCR reaction contain 10 μl 2× SYBR master mix (Roche), 1 μl forward primer (7 μM) and 1 μl reverse primer (7 μM), 1 μl cDNA and 7 μl nuclease-free water. The real-time PCR was performed for 30 cycles on a 7500 Real-Time PCR System (Applied Biosystems, Foster City, CA, USA). Each cycle of real-time PCR includes denaturing for 10 s at 94°C, annealing and extension for 30 s at 58°C. The comparative Ct method was performed to analyze the relative expression of target miRNAs [47]. PCR primer sequences are included in Supplementary Table S1.

### Immunofluorescence assay

MCF-7 and MDA-MB-231 cells were seeded in the flow dish overnight at 37°C and then MCF-7 cells was treated with 4 μM SSA, 30 ng/ml TGFβ1, vehicle control (0.1% DMSO) or combination of 4 μM SSA and 30 ng/ml TGFβ1 for 12 h or pretreated with 4 μM SSA for 12h before adding 30 ng/ml TGFβ1 for 1h. MDA-MB-231 cells were treated with 4 μM SSA or same volume of 0.1% DMSO for 12 h. TNF-α (BD Bioscience) at a concentration of 25 ng/ml was added to the cells for 20 min. After fixation by 4% formaldehyde (Sigma-Aldrich) for 10 min, then the cells were permeabilized with 1% Trition X-100 (Bio-Rad) and blocked with 1% BSA before incubation with Phospho-Smad-2-(Ser465/467)/Smad-3-(Ser423/425) antibody (Cell signaling) and Smad4 antibody (EMD Millipore, Billerica, MA, USA) or NF-κB P65 antibody (BD Bioscience) at 4°C overnight. After washing with PBS, the cells were incubated with the Alexa Fluor^®^ 555-conjugated secondary antibody (Life Technologies) and Alexa Fluor^®^ 647-conjugated secondary antibody (Life Technologies) for 1 h at room temperature. After washing and staining with 5 ng/ml DAPI (Sigma-Aldrich), imaging was taken by using Nikon Eclipse Ti Laser Confocal Scanning Microscopy (Nikon Instruments Inc, Melville, NY, USA). The relative fluorescence intensity of NF-κB and p-Smad2/3 in the nucleus was quantitated by using NIS-Elements AR imaging software (Nikon).

### Chromatin immunoprecipitation assay (ChIP)

ChIP analysis was performed with EZ-Magna ChIP kit (Cat. no. 17-409) from Millipore (Billerica, MA, USA). The procedure strictly followed the manufacturer's instructions. Briefly, 1 × 10^7^ MCF-7 cells were cultured in a 15-cm culture dish and treated with 30 ng/ml TGFβ1 for 1 h before crosslinking by using 1% formaldehyde (Sigma-Aldrich). The fixed cells were lysed, and the chromatin was sheared by sonication using an optimized condition. The chromatin fraction was immunoprecipitated overnight at 4°C with the anti-phosphorylated Smad-3 antibody (Cell signaling), anti-RNA polymerase antibody, and goat-anti-mouse IgG. The DNA was extracted and purified after the immunoprecipitation. PCR amplification was performed in a total volume of 20 μl with pre-designed primers, and the sequences of primers are listed in Supplementary Table S1. PCR reactions were performed for 30 cycles consisting of denaturing for 20s at 94°C, annealing for 30s at 59°C and extension for 30 s at 72°C.

## SUPPLEMENTARY FIGURE AND TABLE


