# Supplementary material for: Inhibition of breast cancer cell motility with a non-cyclooxygenase inhibitory derivative of sulindac by suppressing TGFβ/miR-21 signaling
Source: Oncotarget. 2016 Jan 12;7(7):7979–92. doi: 10.18632/oncotarget.6888 (PMC4884969; doi:10.18632/oncotarget.6888)
Supplement: Supplementary file 1 [file oncotarget-07-7979-s001.pdf]

SUPPLEMENTARY FIGURE AND TABLE

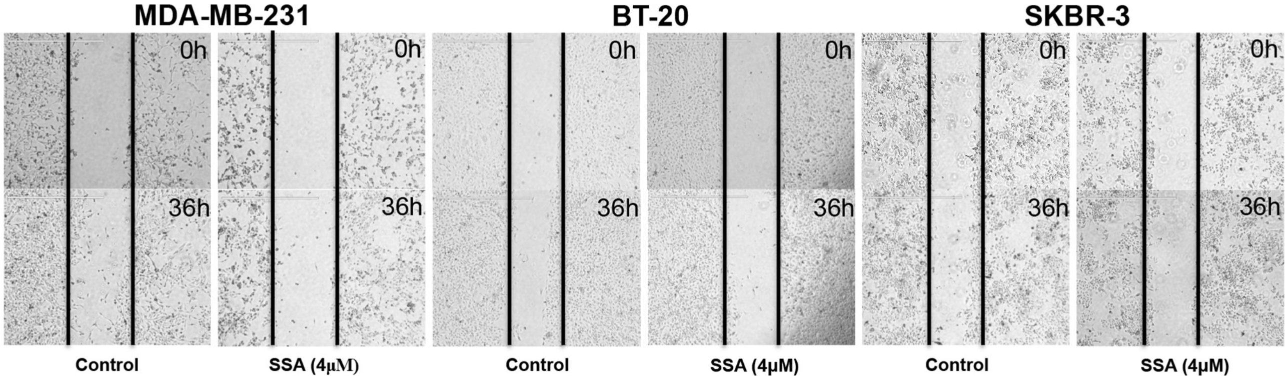

Supplementary Figure S1: SSA inhibits breast cancer cells migration by Wound-Healing assay.

Supplementary Table S1: Primers used in this study

**Primers**		**Primer sequences (5′ to 3′)**
ChIP assay	miR-21 SBS1	Forward: TGAGAAGTCCCACATTTATCACC
		Reverse: AGGGAGGGCAGTTTCTTTTT
	miR-21 SBS2	Forward: GGATGACGCACAGATTGTCCT
		Reverse: AAAAAGAAACTGCCCGCCCT
	GAPDH	Forward: TACTAGCGGTTTTACGGGCG
		Reverse: TCGAACAGGAGGAGCAGAGAGCGA
qRT-PCR	mature miR-9	RT:GTCGTATCCAGTGCAGGGTCCGAGGTATTCGCACTGGATACGACTCATACA
		Forward: TCGGCGTCTTTGGTTATCTAGC
		Reverse: GTCGTATCCAGTGCAGGGTCCGAGGT
	mature miR-10b	RT:GTCGTATCCAGTGCAGGGTCCGAGGTATTCGCACTGGATACGACCACAAA
		Forward: TCGGCGTACCCTGTAGAACCGAAT
		Reverse: GTCGTATCCAGTGCAGGGTCCGAGGT
	mature miR-17	RT:GTCGTATCCAGTGCAGGGTCCGAGGTATTCGCACTGGATACGACCTACCT
		Forward: CAAAGTGCTTACAGTGCA
		Reverse: GTCGTATCCAGTGCAGGGTCCGAGGT
	mature miR-21	RT:GTCGTATCCAGTGCAGGGTCCGAGGTATTCGCACTGGATACGACTCAACA
		Forward: TCGGCGTAGCTTATCAGACTGAT
		Reverse: GTCGTATCCAGTGCAGGGTCCGAGGT
	U6	RT: AAAATATGGAACGCTTCACGAATTTG
		Forward: CTCGCTTCGGCAGCACATATACT
		Reverse: ACGCTTCACGAATTTGCGTGTC
